# Identification and characterization of the nitrate assimilation genes in the isolate of *Streptomyces griseorubens* JSD-1

**DOI:** 10.1186/s12934-014-0174-4

**Published:** 2014-12-10

**Authors:** Haiwei Feng, Yujing Sun, Yuee Zhi, Xing Wei, Yanqing Luo, Liang Mao, Pei Zhou

**Affiliations:** School of Agriculture and Biology, Shanghai Jiao Tong University, Shanghai, 200240 China; Key Laboratory of Urban Agriculture (South), Ministry of Agriculture, Shanghai Jiao Tong University, Shanghai, 200240 China; Bor S. Luh Food Safety Research Center, Shanghai Jiao Tong University, Shanghai, 200240 China

**Keywords:** *Streptomyces griseorubens*, Genome sequencing, Nitrogen metabolism, Nitrate reductase, Nitrite reductase, Glutamine synthetase, Glutamate synthase, Glutamate dehydrogenase

## Abstract

**Background:**

*Streptomyces griseorubens* JSD-1 is a novel actinomycete isolated from soil that can utilize nitrate as its sole nitrogen source for growth and these nitrate assimilation genes active in this biotransformation are expected to be crucial. However, little is known about its genomic or genetic background related to nitrogen metabolism in this isolate. Thus, this study concentrates on identification and characterization of genes involved in nitrate assimilation.

**Results:**

To investigate the molecular mechanism of nitrate metabolism, genome sequencing was performed by Illumina Miseq platform. Then the draft genome of a single linear chromosome with 8,463,223 bp and an average G+C content of 72.42% was obtained, which has been deposited at GenBank under the accession number JJMG00000000. Sequences of nitrate assimilation proteins such as nitrate reductase (EC 1.7.99.4), nitrite reductase (EC 1.7.1.4), glutamine synthetase (EC 6.3.1.2), glutamate synthase (EC 1.4.1.13) and glutamate dehydrogenase (EC 1.4.1.2) were acquired. All proteins were predicted to be intracellular enzymes and their sequences were highly identical to those from their similar species owing to the conservative character. Putative 3D structures of these proteins were also modeled based on the templates with the most identities in the PDB database. Through KEGG annotated map, these proteins proved to be located on the key positions of nitrogen metabolic signaling pathway. Finally, quantitative RT-PCR indicated that expression responses of all genes were up-regulated generally and significantly when stimulated with nitrate.

**Conclusion:**

In this manuscript, we describe the genome features of an isolate of *S. griseorubens* JSD-1 following with identification and characterization of these nitrate assimilation proteins such as nitrate reductase, nitrite reductase, glutamine synthetase, glutamate synthase and glutamate dehydrogenase accounts for the ability to utilize nitrate as its sole nitrogen source for growth through cellular localization, multiple sequence alignment, putative 3D modeling and quantitative RT-PCR. In summary, our findings provide the genomic and genetic background of utilizing nitrate of this strain.

**Electronic supplementary material:**

The online version of this article (doi:10.1186/s12934-014-0174-4) contains supplementary material, which is available to authorized users.

## Background

Nutritionally, physically and biologically, soil is a particularly complex and variable environment, of which the indispensable component is inorganic salts. Among these numerous elements, nitrogen is crucial as it supplies essential nutrient for plants and microorganisms. Nitrogen in soil is existed in the organic or inorganic forms. Generally, the inorganic nitrogen mainly includes nitrate and ammonium. Nitrate is preferentially found in temperate climates, while ammonium is dominant in a number of tropical soil types.

Nitrate is metabolized through various reduction processes in organisms. Nitrate reduction, the most important stages of nitrogen recycle in nature, has various functions as follows: (1) as a source of nitrogen by utilization of NO_3_^−^ (nitrate assimilation); (2) as terminal acceptor of electrons by producing metabolic energy during NO_3_^−^ utilization (nitrate respiration); (3) maintain oxidation reduction balance by dissipating excessed energy (nitrate dissimilation). Totally, three different nitrate reducing systems (Nas, Nar and Nap) have been described in microorganisms [1-4]. Assimilatory nitrate reductases (Nas) are usually cytoplasmic enzymes stimulated by nitrate or nitrite and repressed by ammonium with the character of using either NAD(P)H or ferredoxin as physiological electron donor. Membrane-bounded nitrate reductases (Nar) are mainly involved in anaerobic nitrate respiration and denitrification. Finally, periplasmic dissimilatory reductases (Nap) contribute to redox balancing and aerobic or anaerobic denitrification.

Nitrate assimilation is a key process of nitrogen recycling carried out by higher plants [5,6], algae [7], yeasts [8,9], and bacteria [10,11]. The assimilatory process starts when nitrate is transported into the cell by an active transport system. Nitrate is converted to nitrite with the function of nitrate reductase following with the reduction of nitrite to ammonia and then the conversion of ammonia to glutamine through nitrite reductase and glutamine synthetase. Finally, glutamine is transformed into glutamate by glutamate synthase [12]. Both glutamine and glutamate are the essential substrates for protein synthesis and energy metabolism. For example, glutamate is metabolized into ammonia and α-ketoglutarate with the function of glutamate dehydrogenase.

Up to now, bacterial assimilatory nitrate reductases have been classified into at least three categories according to the number of electron transfer cofactors bounded by the catalytic bis-molybdopterin guanine dinucleotide subunit and the nature of the probable electron donors to each enzyme, which are typified by their sources of *Synechococcus* sp. [13], *Klebsiella oxytoca* [14], and *Bacillus* sp. [4,15].

Streptomycetes are a genus of Gam-positive bacteria that form filamentous mycelium-like eukaryote fungi. They are the most numerous and ubiquitous soil bacteria, which are crucial in the environment because of their broad ranges of metabolic processes and biotransformations. To our knowledge, genetic basic related to the metabolism of nitrate in streptomycetes has rarely been reported.

In this study, genome sequencing of our recently isolated soil strain *Streptomyces griseorubens* JSD-1 that could utilize nitrate as the unique nitrogen source for growth, was carried out for identification and characterization of genes participating in the nitrate metabolism.

## Results and discussion

### Identification and characterization of the isolate

A novel strain, designated as JSD-1, was isolated on the basal medium owing to its ability to grow efficiently upon the medium with the addition of nitrate as the unique nitrogen source. Through the combination of 16S rRNA sequencing as well as its morphological and physiological characters, the isolate was identified as *S. griseorubens* [16].

**Figure 1:**
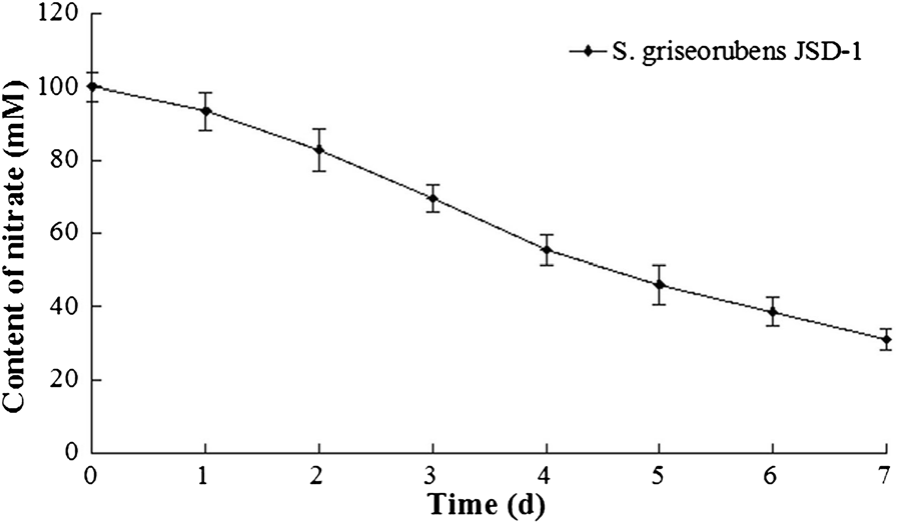
**Nitrate metabolic ability of S. griseorubens JSD-1.**

In addition, *S. griseorubens* JSD-1 could metabolized 69% nitrate totally during the whole cultivation (Figure 1), indicating that this isolate had significant tolerance and metabolic capability towards nitrate of high concentration (100 mM).

### Genome sequencing and bio-informatics analysis of *S. griseorubens* JSD-1

Genome sequencing of *S. griseorubens* JSD-1 was performed by Illumina Miseq 2 × 250 bp platform by Personal biotechnology Co., Ltd. A total of 6,432,848 reads including up to 2,209 Mb clean data were generated, which represented a 263.0-fold average coverage of the whole genome. The assembled genome contained 2 scaffolds and 246 contigs. The N_50_ length of contigs was 53,294 bp and that of scaffolds was 7,563,100 bp. Finally, we obtained the draft genome map of *S. griseorubens* with a single linear chromosome of 8,463,223 bp, which was of smaller size than the ones of *S. coelicolor* A3(2) (8.7 Mb), *S. avermitilis* MA-4680 (9.0 Mb), or *S. griseus* IFO 13350 (8.5 Mb) [17-19]. Besides, the G+C content of was high up to 72.42%, a typical character of streptomycetes [17-22]. Analysis of the genome revealed that it contained 7159 protein-coding sequences (CDS). Among these CDSs, 4587 proteins could be assigned to clusters of orthologous groups (COG) families [23].

Genome features were shown in Table 1, which has been deposited in GenBank under accession number **JJMG00000000**. Since successfully obtaining its draft genome, emphasis was put on identification and characterization of these nitrate assimilation genes.

**Table 1:** **Genome features of** ***S. griseorubens*** **JSD-1**

**Attribute**	**Value**
Total number of contigs	246
Total bases in contigs (bp)	7,600,227
Longest contig length (bp)	328,776
N_20_ length (bp)	104,101
N_50_ length (bp)	53,294
N_90_ length (bp)	16,099
Sequences greater than 1 kb	243
Average coverage	263
DNA G+C content (%)	72.42
Protein coding genes	7,159
Genes with predicted function	4,587

### Cellular localization

No natural signal peptide sequence could be judged from these analyzed proteins on the N-terminals (Additional file 1: Figure S1). Therefore, all nitrate assimilation proteins were predicted to be intracellular enzymes, which was accordance with previous reports [10-12].

### Multiple sequence alignment

After obtaining the assembled genome, genes were predicted and annotated by BLASTP to find out the nitrate assimilation genes. Therefore, genes found were nitrate reductase electron transfer subunit NarB (2394 bp), nitrate reductase catalytic subunit NarC (2127 bp), nitrite reductase [NAD(P)H] large subunit NirD (2601 bp), nitrite reductase [NAD(P)H] small subunit NirE (354 bp), glutamine synthetase GlnA (1377 bp), glutamate synthase large subunit GltF (4512 bp), glutamate synthase small subunit GltG (1491 bp), and glutamate dehydrogenase GdhH (4950 bp). Since protein sequences were obtained. Searches against the Nr database were performed to find out the top three sequences with the most identities for the following multiple sequence alignments.

**Figure 2:**
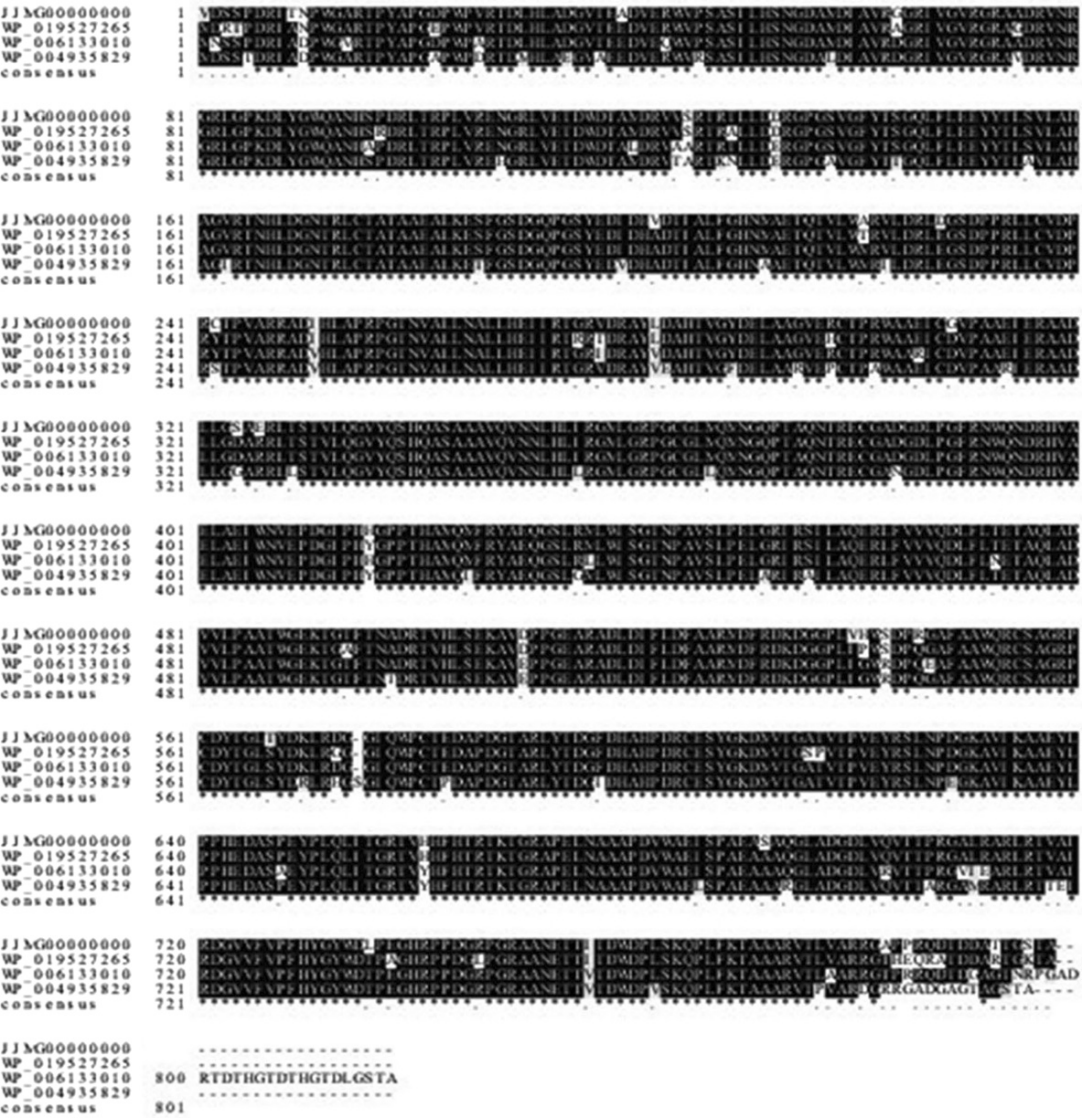
**Multiple sequence alignment of nitrate reductase electron transfer subunit (NarB) from its similar species.**

As nitrate reductase, nitrite reductase, and glutamate synthase were dimer proteins consisting of two subunits, NarB, NirD, GlnA, GltG, and GdhH were used to represent the five analyzed proteins for further study. Results in Table 2 were summarized as follows: (1) the top three proteins with the most identities to nitrate reductase electron transfer subunit (NarB) were from *Streptomyces* sp. FxanaD5 (WP_019527265) of 95% identity, *S. gancidicus* (WP_006133010) of 94% identity, and *S. griseoflavus* (WP_004935829) of 89% identity; (2) the top three proteins with the most identities to nitrite reductase large subunit (NirD) were from *Streptomyces* sp. FxanaD5 (WP_019523194) of 97% identity, *S. chartreusis* (WP_010042208) of 94% identity, and *S. gancidicus* (WP_006129667) of 94% identity; (3) the top three proteins with the most identities to glutamine synthetase (GlnA) were from *Streptomyces* sp. FxanaD5 (WP_019522401), *S. gancidicus* (WP_006130087), and *S. griseoflavus* (WP_004931998) with the same identity of 98%; (4) the top three proteins with the most identities to glutamate synthase small subunit (GltG) were from *Streptomyces* sp. FxanaD5 (WP_019525300) following *S. gancidicus* (WP_006135305) and *Streptomyces* sp. Amel2xE9 (WP_019984065) with the identities of 95%, 94%, and 88%, respectively; (5) the top three proteins with the most identities to glutamate dehydrogenase (GdhH) were from *A. acidiphila* (WP_033277556), *Streptomyces* sp. NRRL S-1314 (WP_031019546), and *S. gancidicus* (WP_006132432) with the identities of 99%, 96%, and 96%, respectively. Refer to Figure 2 for the visible alignment of nitrate reductase electron transfer subunit (NarB) and other results were also exhibited (Additional file 2: Figure S2; Additional file 3: Figure S3; Additional file 4: Figure S4; Additional file 5: Figure S5).

**Table 2:** **Multiple sequence alignments of nitrate assimilation proteins**

**Proteins**	**Significant alignments (Accession number)**	**Alignment identifies (%)**
NarB	*Streptomyces* sp. FxanaD5 (WP_019527265)	756/797 (95%)
	*Streptomyces gancidicus* (WP_006133010)	745/793 (94%)
	*Streptomyces griseoflavus* (WP_004935829)	712/799 (89%)
NarC	*Streptomyces gancidicus* (WP_006129675)	670/708 (95%)
	*Streptomyces griseoflavus* (WP_004931583)	622/694 (90%)
	*Streptomyces viridosporus* (WP_016826372)	632/722 (88%)
NirD	*Streptomyces* sp. FxanaD5 (WP_019523194)	839/866 (97%)
	*Streptomyces chartreusis* (WP_010042208)	814/862 (94%)
	*Streptomyces gancidicus* (WP_006129667)	821/869 (94%)
NirE	*Streptomyces gancidicus* (WP_006129666)	111/117 (95%)
	*Streptomyces* sp. FxanaD5 (WP_019523193)	107/117 (91%)
	*Streptomyces sviceus* (WP_007381873)	94/117 (80%)
GlnA	*Streptomyces* sp. FxanaD5 (WP_019522401)	449/458 (98%)
	*Streptomyces gancidicus* (WP_006130087)	444/453 (98%)
	*Streptomyces griseoflavus* (WP_004931998)	443/453 (98%)
GltF	*Streptomyces* sp. FxanaD5 (WP_019525478)	1477/1503 (98%)
	*Streptomyces gancidicus* (WP_006135499)	1470/1508 (97%)
	*Streptomyces ghanaensis* (WP_004989383)	1414/1503 (94%)
GltG	*Streptomyces* sp. FxanaD5 (WP_019525300)	471/495 (95%)
	*Streptomyces gancidicus* (WP_006135305)	467/496 (94%)
	*Streptomyces* sp. Amel2xE9 (WP_019984065)	436/496 (88%)
GdhH	*Actinospica acidiphila* (WP_033277556)	1644/1649 (99%)
	*Streptomyces* sp. NRRL S-1314 (WP_031019546)	1585/1648 (96%)
	*Streptomyces gancidicus* (WP_006132432)	1566/1630 (96%)

Since NarB, NirD, GlnA, GatG, and GdhH shared high identities with sequences of their similar strains in the primary structure, especially *S. gancidicus*, *Streptomyces* sp*.* FxanaD5, and *S. griseoflavus*, their sequences were inferred to be conservative during evolution. However, nitrite reductase [NAD(P)H] small subunit (NirD) seemed to be only identical with *S. gancidicus* and *Streptomyces* sp. FxanaD5 as the identities dropped to 80% when aligned with other streptomycetes. It could be either the sequence was not universally functional annotated or it had other special functions [24], which needed to be further investigated.

### Putative 3D structure modeling

After obtaining the sequences of these nitrate assimilation proteins (nitrate reductase, nitrite reductase, glutamine synthetase, glutamate synthase, and glutamate dehydrogenase), their putative 3D structures were modeled by PHYRE2 based on the most identical templates in the PDB database. By viewing the modeling results, a brief idea of how the 3D structures of these proteins might look like would be illustrated (Additional file 6: Figure S6).

All details were shown in Table 3. Putative 3D structure modelings of (1) NarB was generated based on the sequence residues from 37 to 777 with 89% coverage and 33% identity against the template c2v45A (2.40 Å); (2) NirD was generated based on the sequence residues from 5 to 413 with 47% coverage and 26% identity against the template c3ntaA (2.01 Å); (3) GlnA was generated based on the sequence residues from 4 to 452 with 96% coverage and 35% identity against the template c1fpyE (2.89 Å); (4) GltG was generated based on sequence residues of 5 to 493 with 94% coverage and 21% identity against the template c1gthD (2.25 Å); (5) GdhH was generated based on sequence residues of 732 to 1284 with low coverage and identity of 33% and 18% against the template c1hrdA (1.96 Å). All the predictions above were of 100% confidence with the modeling results.

**Table 3:** **Putative 3D modeling information of nitrate assimilation proteins**

**Proteins**	**Residue range (AA)**	**Query coverage (%)**	**Identity (%)**	**Confidence (%)**	**Template**
NarB	37-777	89%	33%	100%	c2v45A (2.40 Å)
NarC	1-705	99%	34%	100%	c2v45A (2.40 Å)
NirD	5-413	47%	26%	100%	c3ntaA (2.01 Å)
NirE	18-116	85%	51%	100%	c4aivA (2.00 Å)
GlnA	4-452	96%	35%	100%	c1fpyE (2.89 Å)
GltF	11-1477	96%	46%	100%	c2vdcF (9.50 Å)
GltG	5-493	94%	21%	100%	c1gthD (2.25 Å)
GdhH	732-1284	33%	18%	100%	c1hrdA (1.96 Å)

Among these modelings, NirD, GltG, and GdhH shared low (below 30%) identities of 26%, 21%, and even 18% with the templates although they all shared high similarities with their identical sequences in the primary structure (Table 2). It could be either there were not enough identical protein templates in the database or these proteins had rarely secondary structures [25]. Although the overall identities to the modeling templates were discriminating, it simply offered a brief demonstration of these proteins’ 3D structures with 100% confidence, which indicated that all predictions were reliable and accurate.

### Location of nitrate assimilation proteins in KEGG metabolic pathway

**Figure 3:**
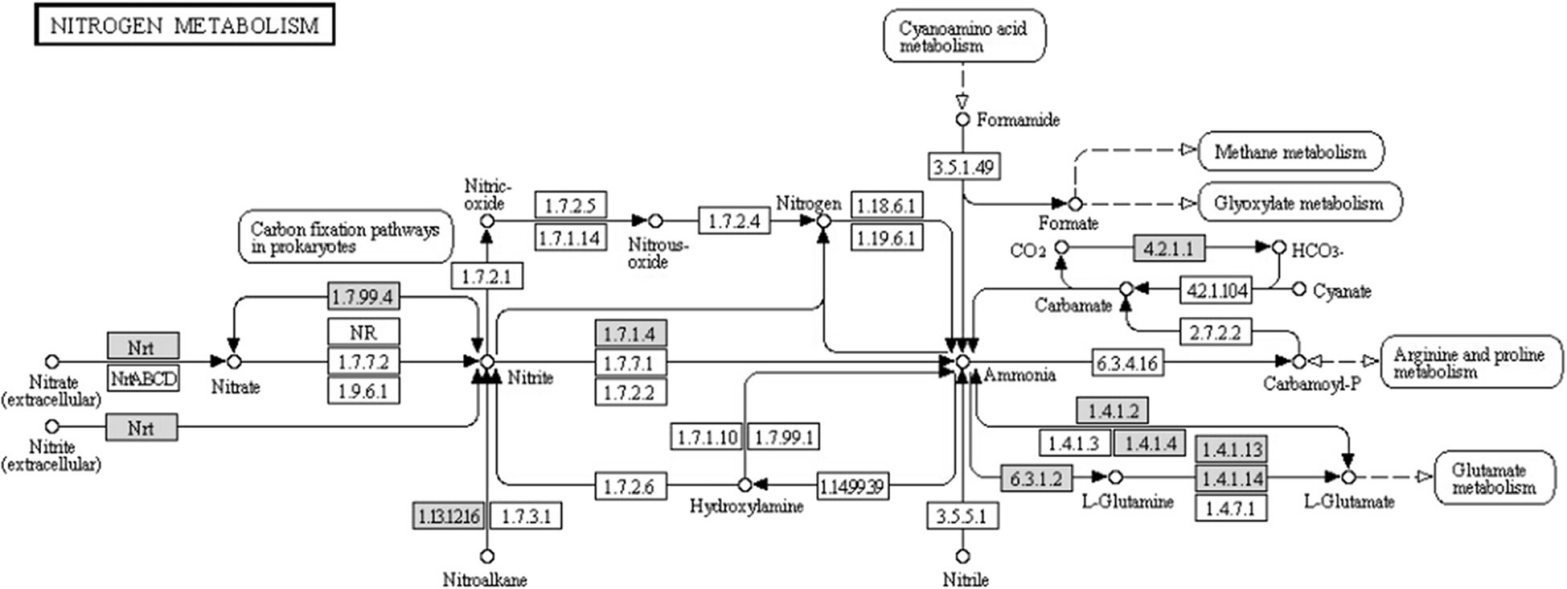
**Enzymes involved in the nitrogen metabolic signaling pathway from KEGG annotation.** Genes marked with grey represent the existence in this species.

To investigate the location of these nitrate assimilation proteins in nitrogen metabolic pathway, a search was carried out in the KEGG database. Inferred from the KEGG map (Figure 3), nitrate reductase (EC 1.7.99.4), nitrite reductase (EC 1.7.1.4), glutamine synthetase (EC 1.3.1.2), glutamate synthase (EC 1.4.1.13) and glutamate dehydrogenase (EC 1.4.1.2) were located on the key positions of nitrogen metabolic pathway. The pathway could be summarized as follows: nitrate was transported into the cell through an active transport system following the reduction of nitrate to ammonium by nitrate reductase and nitrite reductase. Combined with glutamine acid, glutamine was formed by glutamine synthetase and then glutamine was converted into glutamate with the function of glutamate synthase [12]. Finally, glutamate was metabolized into ammonia and α-ketoglutarate by glutamate dehydrogenase. Both glutamine and glutamate could be directly utilized for protein bio-synthesis and other biochemistry processes such as tricarboxylic acid cycle.

### Differential expression patterns of the analyzed proteins

To further investigate the impact of nitrate concentrations (10 mM, 30 mM, 50 mM, and 100 mM) and cultivation duration on the expression patterns of nitrate assimilation proteins, quantitative RT-PCR was performed. Generally, expression of nitrate reductase and nitrite reductase were up-regulated with the concentrations of nitrate increasing. However, expression patterns of glutamine synthetase and glutamate synthase reached the highest when stimulated by 50 mM nitrate. It was inferred that mass of glutamine or glutamate newly formed that beyond the capacity of biosynthesis were accumulated through nitrate metabolic pathway, and glutamine or glutamate excessed led to the substrate feedback inhibition [26]. Thereafter, expression levels of glutamine synthetase and glutamate synthase were down-regulated. Glutamate dehydrogenase exhibited the similar tendency of expression with the peak concentration of 30 mM nitrate for the mass of exceed ammonia sourced from the nitrite reduction and glutamate dehydrogenation. However, expression patterns of nitrate reductase or nitrite reductase were still up-regulated even when nitrate reached up to 100 mM. As nitrate or nitrite of high concentrations were extremely poisonous to cells, bio-transforming nitrate or nitrite immediately was urgently needed [27]. It was supposed to be the genetic basic accounts for its significant tolerance towards nitrate or nitrite of high concentrations.

**Figure 4:**
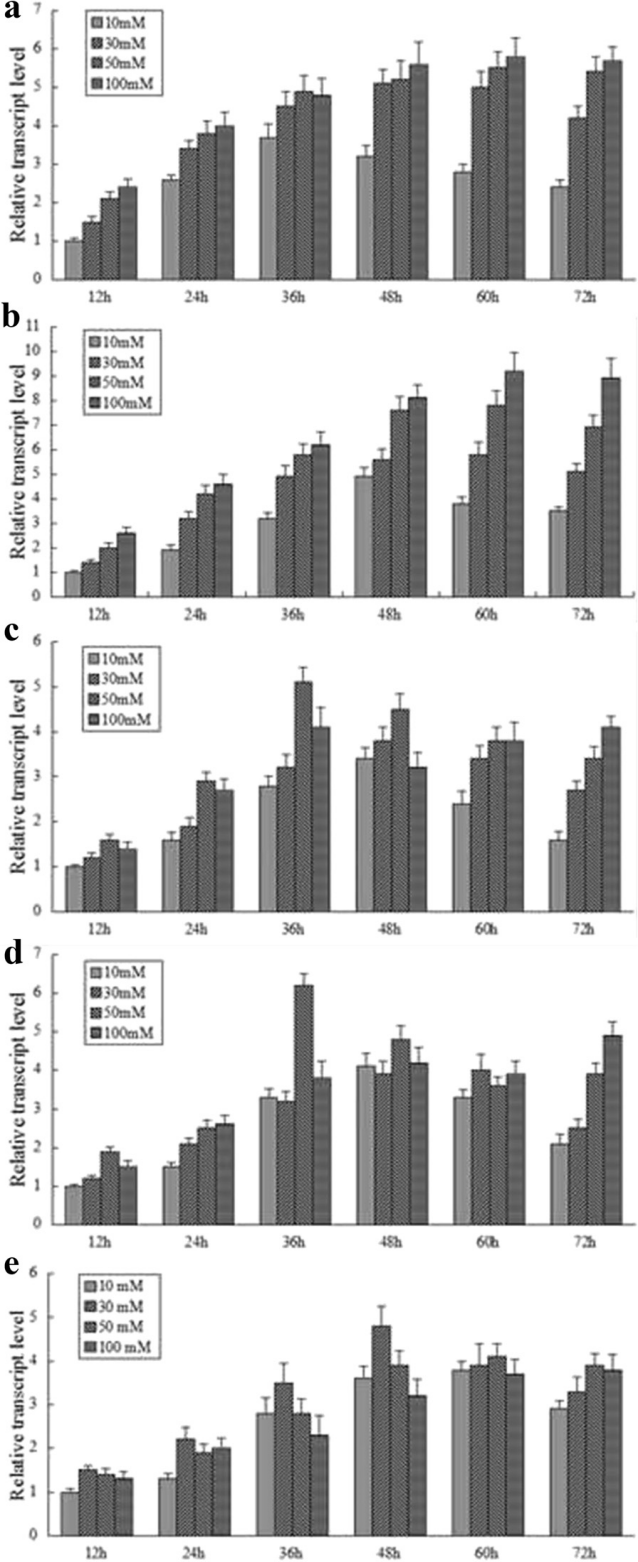
**Differential expression patterns of the analyzed proteins when stimulated with nitrate.** Figures are noted as **a** nitrate reductase, **b** nitrite reductase, **c** glutamine synthetase, **d** glutamate synthase, **e** glutamate dehydrogenase.

Results also clearly demonstrated that cultivation duration had significant influence on the expression patterns of these analyzed proteins. When exposed to nitrate of low concentrations (10 mM or 30 mM), expression responses of these proteins showed a consistent tendency of rising firstly and then falling with the peaks occurring at around 48 h or 60 h. Expression levels were down regulated due to the lack of substrate after certain cultivation period [28,29]. With concentrations of nitrate rising, expression of nitrate reductase and nitrite reductase exhibited an rising tendency. However, expression of glutamine synthetase, glutamate synthase, and glutamate dehydrogenase showed an identical tendency of rising firstly and then falling owing to the substrate feedback inhibition referred. Refer to Figure 4 for the detailed results.

## Conclusions

In this manuscript, we obtained the draft genome map of *S. griseorubens* JSD-1, a novel actinomycete that could utilize nitrate as its sole nitrogen source for growth by Illumina Miseq platform. Through BLASTP searches against Nr protein database, proteins involved in nitrate assimilation were picked out. All analyzed proteins were predicted to be intracellular enzymes and sequence alignments showed that these protein sequences were highly identical to those of their similar species. The same conclusion could also be drawn from their 3D structure modelings, both indicating the conservative character from their primary and secondary structures.

Since these analyzed proteins were determined to be located on the key positions of nitrogen signaling pathway, nitrate metabolism was inferred and summarized as: nitrate extracellular was transported into cells by membrane transporters. Reduction of nitrate to ammonia through assimilatory nitrate reductase and nitrite reductase were followed. Finally, ammonium was incorporated into carbon skeletons by glutamine synthetase, glutamate synthetase and glutamate dehydrogenase. Besides, expression patterns of these proteins were investigated by quantitative RT-PCR. Generally, nitrate reductase and nitrite reductase were up-regulated expressed when stimulated by nitrate. However, expression levels of glutamine synthetase, glutamate synthase or glutamate dehydrogenase reached the highest when the concentration of nitrate reached the threshold values (30 mM or 50 mM) which then led to the substrate feedback inhibition. Moreover, cultivation duration also had significant influence on expression responses of these nitrate assimilation proteins, with a consistently tendency of rising firstly and then falling for the shortage of substrate.

## Materials and methods

### Isolation, identification and characterization of the isolate

The isolate was sourced from soil collected from Shanghai, China on a defined basal solid medium (20.0 g/L glucose, 10.0 g/L KNO_3_, 1.0 g/L MgSO_4_, 0.5 g/L KH_2_PO_4_, 0.5 g/L NaCl, 0.25 g/L CaCl_2_, 0.01 g/L FeSO_4_·7H_2_O, 15.0 g/L agar, pH 7.0). The strain was maintained on nutrient agar slants and then sun-cultured periodically.

Genome DNA was extracted and purified from 2.5 ml YEME cultures (3.0 g/L yeast extract, 5.0 g/L tryptone, 3.0 g/L malt extract, 10.0 g/L glucose, and 340.0 g/L sucrose, natural pH) at 32°C for 48 h using Bacteria Genomic DNA Extraction Kit (TIANGEN, Beijing, China) for strain identification and genome sequencing. PCR amplification of 16S rRNA gene of the isolate was performed and sequence obtained was used to search against the NCBI database (http://www.ncbi.nlm.nih.gov). In addition, morphological and physiological characteristics were also determined for identification.

To determine the capability of nitrate metabolism, nitrate content remained in the basal liquid medium (100 mM KNO_3_) were sampled and measured every 24 h during 7 days’ cultivation at 32°C according to the instruction of AutoAnalyzer 3 (SEAL Analytical, Ltd). Technically triplicates were performed for quantification.

### Genome sequencing and functional annotation

The genome sequencing of JSD-1 was performed by Illumina MiSeq 2×250 bp platform with insert sizes of 300 bp, 360 bp, and 700 bp paired-end as well as 3 kb and 8 kb mate-paired libraries. Assembly of all sequence reads applying Newbler 2.8 assembler resulted in a draft genome. Glimmer 3.0 were used to predict open reading frames (ORFs) with BLASTP annotation [30,31]. The functional annotation was determined with the KEGG, COG, and Swiss-Prot databases [32-34]. Finally, the draft genome map of the *S. griseorubens* JSD-1 was submitted to the GenBank for further investigation.

### Cellular localization

After obtaining sequences of these nitrate assimilation proteins, their cellular localizations as well as signal peptide cleavage sites, were predicted by the online program SignalP 4.1 in fasta format [35].

### Multiple sequence alignment

After comparing sequences of these nitrate assimilation proteins with the Nr database by BLASTP, the top three sequences with the most identities were picked out for sequence alignments. Alignments were performed by program ClustalX (Version 1.83) then results were further polished by the online program BoxShade (Version 3.21).

### Putative 3D structure modelings

Putative 3D structures of these analyzed proteins were predicted by online program PHYRE 2.0 Server (http://genome3d.eu/) in the intensive mode [36]. Protein sequences were used to search against the ExPDB template library. Sequences with the most identities were used for model generation. All 3D images were generated in JSmol colored by rainbow from N to C terminals [37].

### Expression patterns of the analyzed genes by quantitative RT-PCR

Cultures collected from the basal medium with different concentrations (10 mM, 30 mM, 50 mM, or 100 mM) of KNO_3_ were used to extract purified RNA. Total RNAs of *S. griseorubens* JSD-1 were obtained according to RNAprep Pure Bacteria Kit (TIANGEN, Beijing, China). Cells were sampled every 12 h within 3 days. Primers of these analyzed genes as well as 16S rRNA gene were designed by DNAMAN 6.0. The details of primers and PCR products were listed in Table 4.

**Table 4:** **Primers used for quantitative RT-PCR**

**Gene**	**Primer (5′-3′)**	**Target location**	**PCR size (bp)**
**16S rRNA**	F: CGTATTCACCGCAGCAATGC	94-179	86
	R: GCGAGGTGGAGCGAATCTCA		
**NarB**	F: GACCTCTACGGCTGGCAGGC	259-402	144
	R: GCGGTCGTCAAGCAGGGT		
**NirD**	F: GCGTGGTCGTGCTGTGCGA	101-207	107
	R: CGCCAGGTCCGTCAGCGACA		
**GlnA**	F: GCTGAGCCTGATGGAACGCA	1227-1322	96
	R: GCCTCCCACTCCTGCTTCT		
**GltG**	F: CGGTGTGCCGTTCTGTCA	150-269	120
	R: AAGTTGTTCGTGGCGTGC		
**GdhH**	F: AAACTGCCGACTGGGACC	82-189	108
	R: GCGGTCGGTGAGGTCTTC		

The reverse transcription PCR for cDNA was performed on the ABI StepOne™ sequence detection system (PE Applied Biosystems, Foster City, USA). Quantitative RT-PCR was performed using the SYBR Green dye method according to the manufacturer’s recommendations of SYBR® Premix Ex Taq™ GC (TaKaRa, Dalian, China). Cycling parameters of quantitative RT-PCR reactions were programmed with an initial step of 30 s at 95°C followed by 40 cycles consisting of denaturation at 95°C for 10 s, annealing at 60°C for 30 s and extension at 72°C for 15 s. The relative quantification of these genes was analyzed with the 2^-**∆∆**Ct^ method. Technical triplicates were performed for each biological replicate, and the average values were used for quantification. Herein 16S rRNA was used as internal control to normalize the relative transcription of these analyzed genes.

## Additional files

Additional file 1: Figure S1. Cellular localization of nitrate assimilation proteins. a nitrate reductase electron transfer subunit (NarB), b Nitrite reductase large subunit (NirD), c Glutamine synthetase (GlnA), d Glutamate synthase small subunit (GltG), e Glutamate dehydrogenase (GdhH). Additional file 2: Figure S2. Multiple sequence alignment of nitrite reductase large subunit (NirC) from its similar species. Additional file 3: Figure S3. Multiple sequence alignment of glutamine synthetase (GlnE) from its similar species. Additional file 4: Figure S4. Multiple sequence alignment of glutamate synthase small subunit (GltG) from its similar species. Additional file 5: Figure S5. Multiple sequence alignment of glutamate dehydrogenase (GdhH) from its similar species. Additional file 6: Figure S6. Putative 3D structure modelings of nitrate assimilation proteins. a Nitrate reductase electron transfer subunit (NarB), b Nitrite reductase large subunit (NirD), c Glutamine synthetase (GlnA), d Glutamate synthase small subunit (GltG), e Glutamate dehydrogenase (GdhH).
