# Supplementary figures and images for: Identification and characterization of the nitrate assimilation genes in the isolate of Streptomyces griseorubens JSD-1
Source: Microb Cell Fact. 2014 Dec 10;13:174. doi: 10.1186/s12934-014-0174-4 (PMC4272520; doi:10.1186/s12934-014-0174-4)

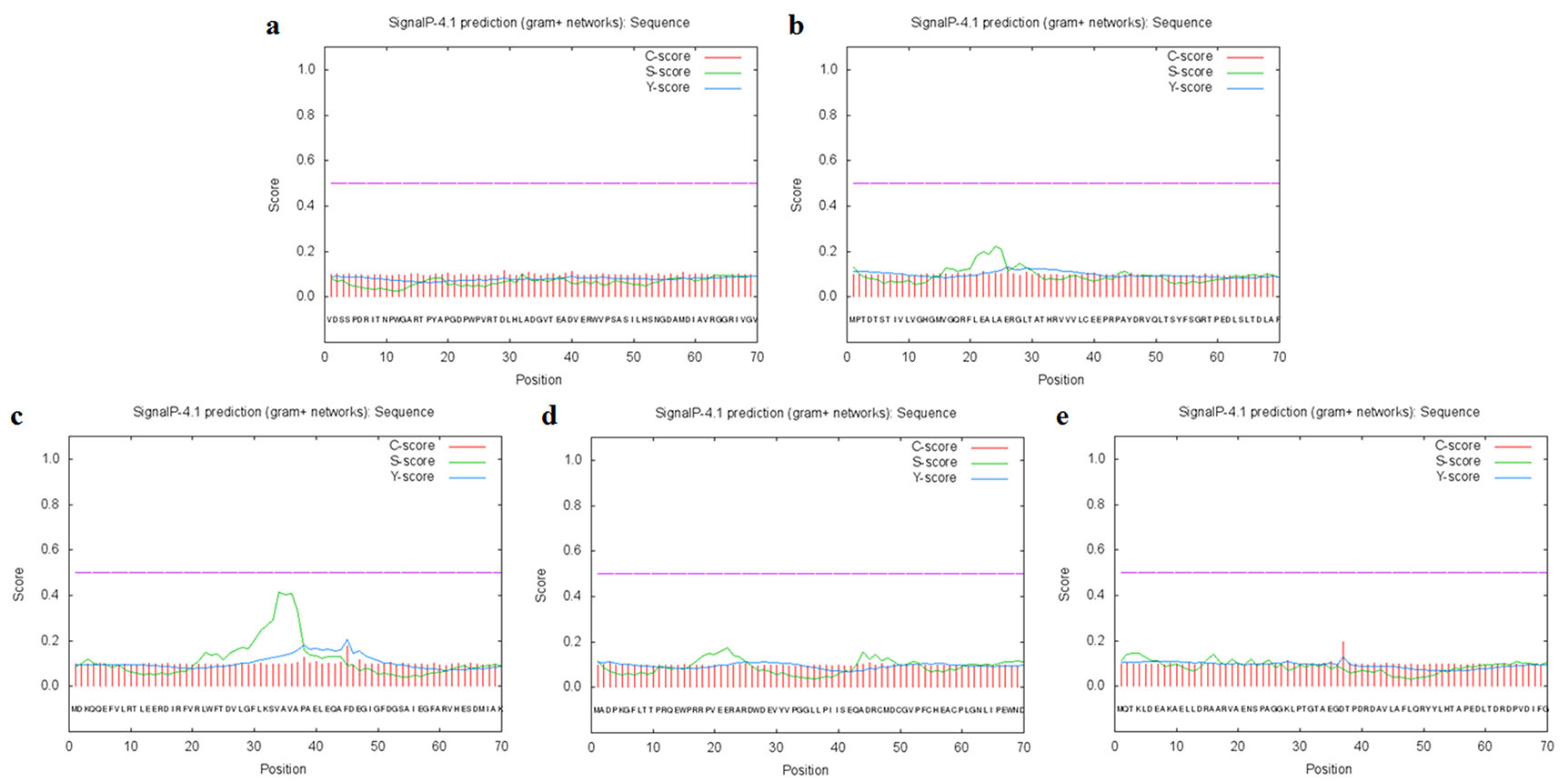

Supplement: Additional file 1: Figure S1. — Cellular localization of nitrate assimilation proteins. a nitrate reductase electron transfer subunit (NarB), b Nitrite reductase large subunit (NirD), c Glutamine synthetase (GlnA), d Glutamate synthase small subunit (GltG), e Glutamate dehydrogenase (GdhH). [file 12934_2014_174_MOESM1_ESM.jpeg]

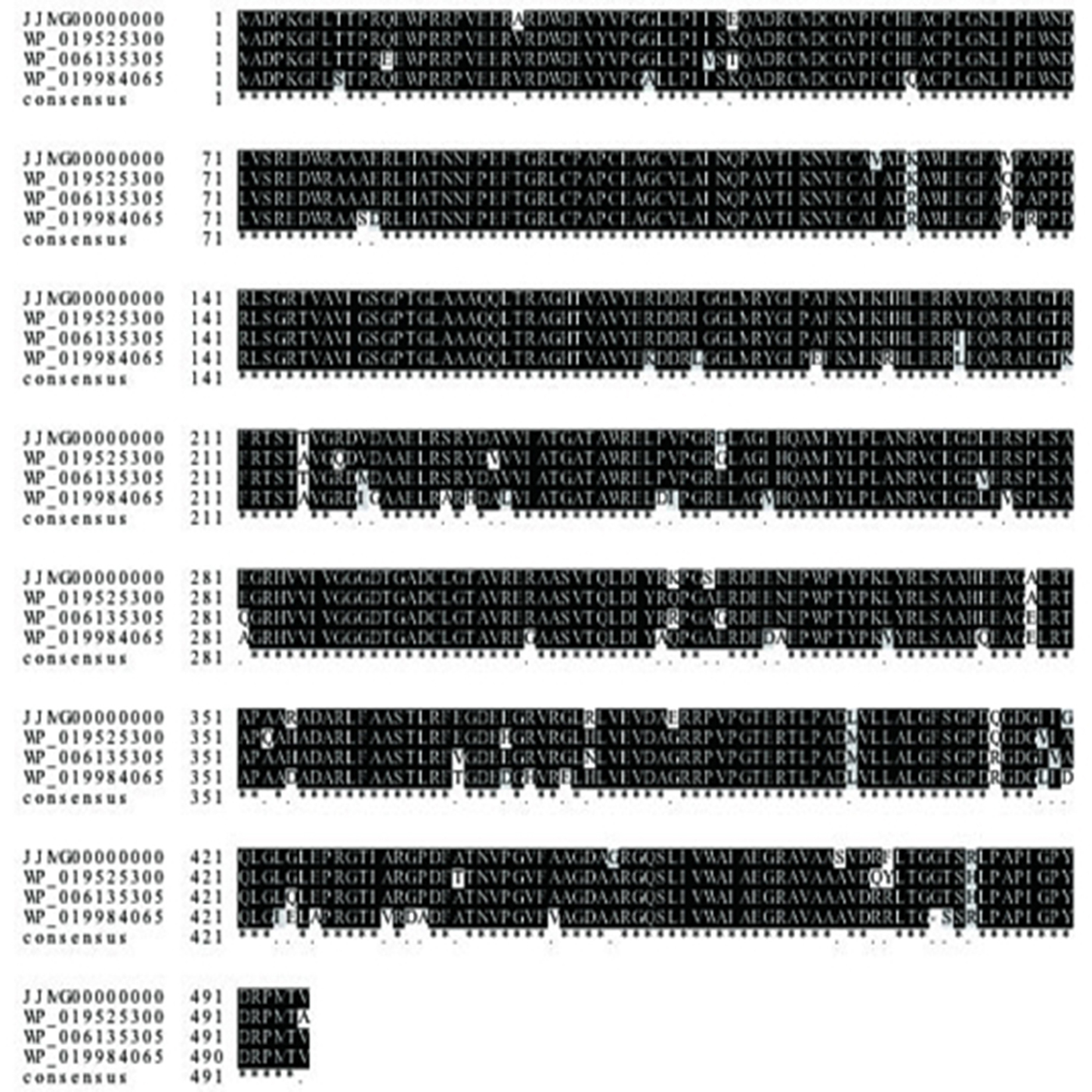

Supplement: Additional file 2: Figure S2. — Multiple sequence alignment of nitrite reductase large subunit (NirC) from its similar species. [file 12934_2014_174_MOESM2_ESM.jpeg]

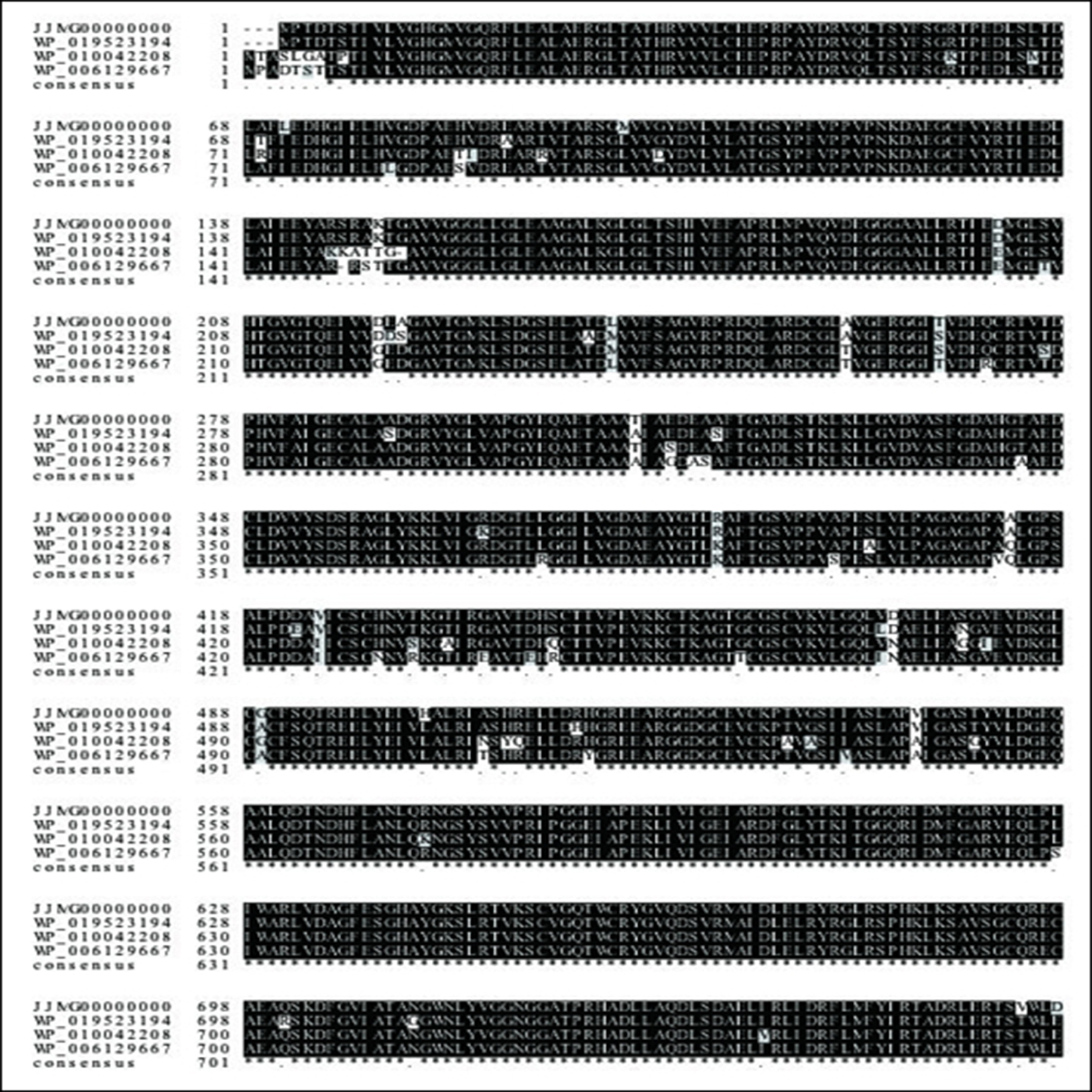

Supplement: Additional file 3: Figure S3. — Multiple sequence alignment of glutamine synthetase (GlnE) from its similar species. [file 12934_2014_174_MOESM3_ESM.jpeg]

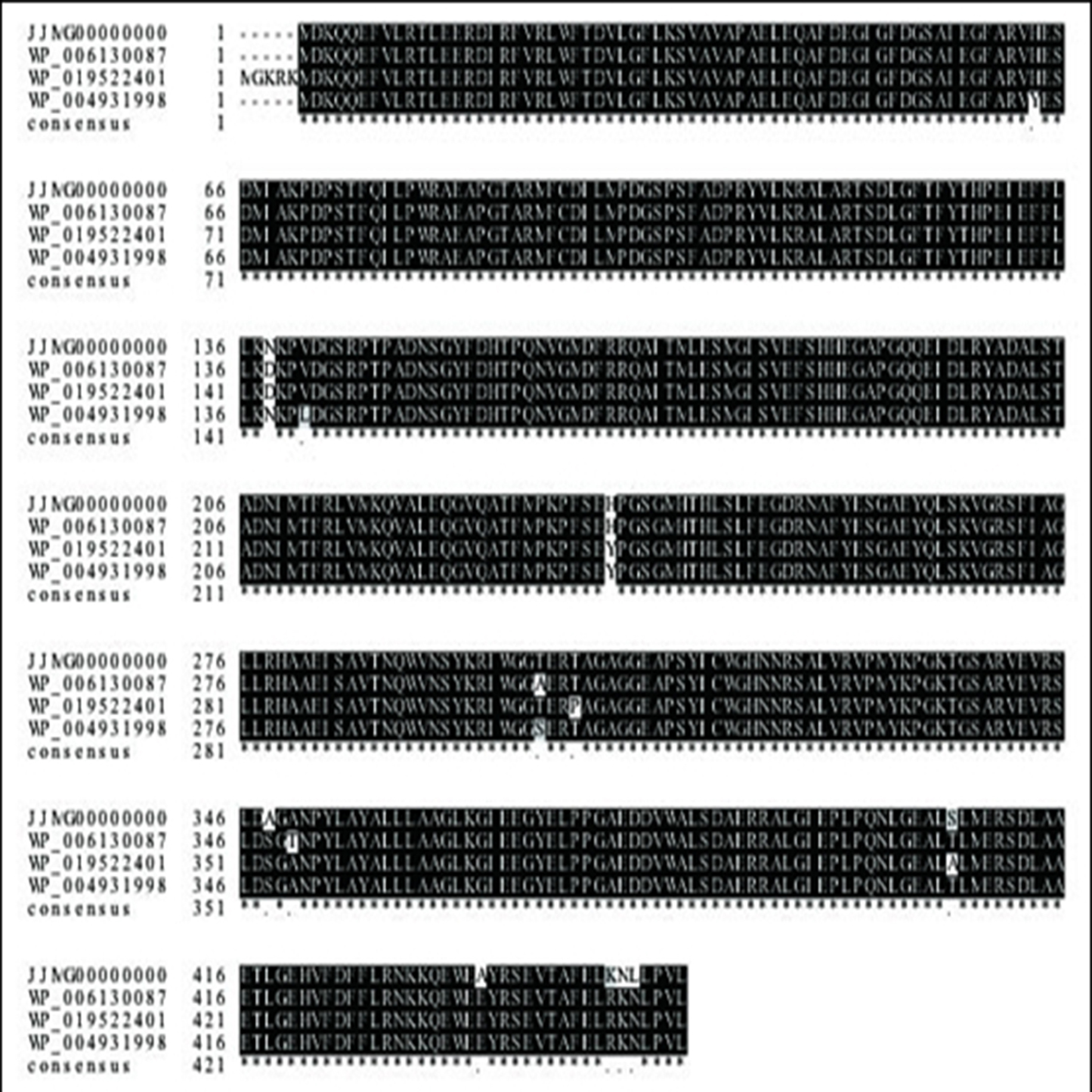

Supplement: Additional file 4: Figure S4. — Multiple sequence alignment of glutamate synthase small subunit (GltG) from its similar species. [file 12934_2014_174_MOESM4_ESM.jpeg]

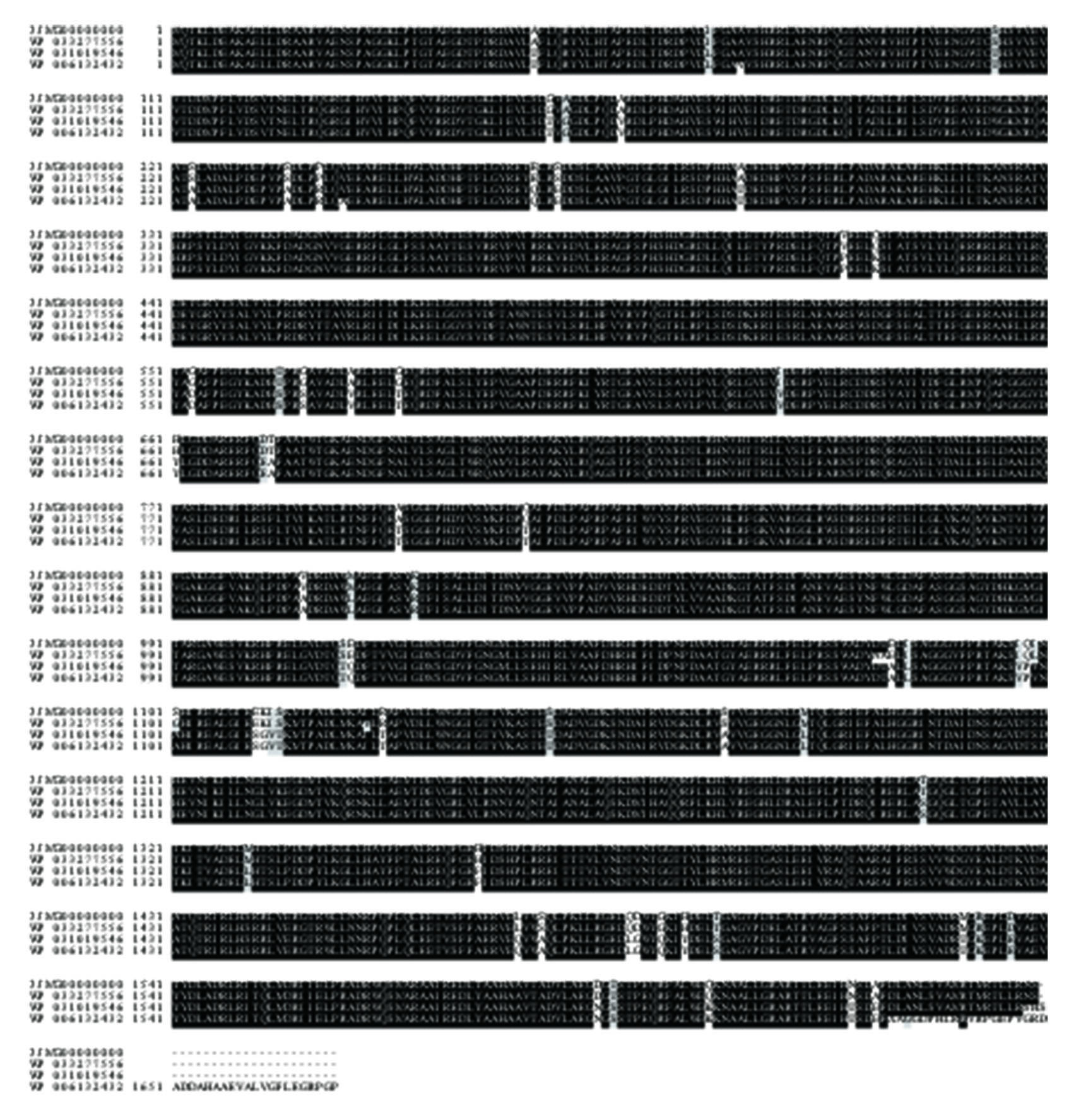

Supplement: Additional file 5: Figure S5. — Multiple sequence alignment of glutamate dehydrogenase (GdhH) from its similar species. [file 12934_2014_174_MOESM5_ESM.jpeg]

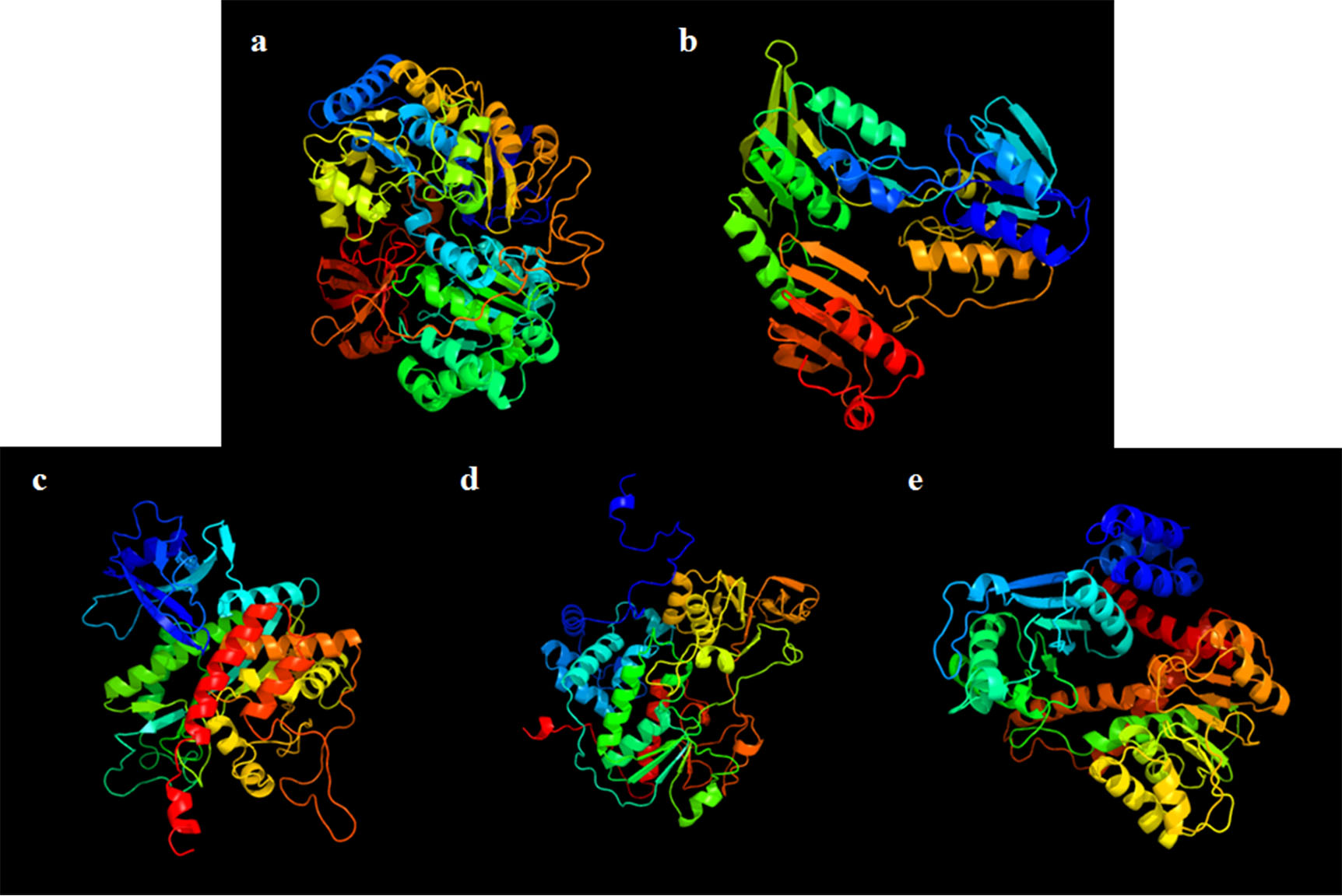

Supplement: Additional file 6: Figure S6. — Putative 3D structure modelings of nitrate assimilation proteins. a Nitrate reductase electron transfer subunit (NarB), b Nitrite reductase large subunit (NirD), c Glutamine synthetase (GlnA), d Glutamate synthase small subunit (GltG), e Glutamate dehydrogenase (GdhH). [file 12934_2014_174_MOESM6_ESM.jpeg]
